# Proteogenomic characterization and integrative analysis of glioblastoma multiforme

**DOI:** 10.18632/oncotarget.21937

**Published:** 2017-10-19

**Authors:** Ying-Chun Song, Gai-Xia Lu, Hong-Wei Zhang, Xiao-Ming Zhong, Xian-Ling Cong, Shao-Bo Xue, Rui Kong, Dan Li, Zheng-Yan Chang, Xiao-Feng Wang, Yun-Jie Zhang, Ran Sun, Li Chai, Ru-Ting Xie, Ming-Xiang Cai, Ming Sun, Wei-Qing Mao, Hui-Qiong Yang, Yun-Chao Shao, Su-Yun Fan, Ting-Miao Wu, Qing Xia, Zhong-Wei Lv, David A. Fu, Yu-Shui Ma

**Affiliations:** ^1^ Department of Nuclear Medicine, Shanghai Tenth People’s Hospital, Tongji University School of Medicine, Shanghai 200072, China; ^2^ Institute of Health Sciences, Shanghai Institutes for Biological Sciences, Chinese Academy of Sciences, Shanghai 200025, China; ^3^ Department of Radiology, Jiangxi Provincial Tumor Hospital/Ganzhou City People’s Hospital, Nanchang 330029, China; ^4^ Department of Biobank, China-Japan Unoin Hospital, Jilin University, Changchun 130033, China; ^5^ Department of Pathology, Shanghai Tenth People’s Hospital, Tongji University School of Medicine, Shanghai 200072, China; ^6^ Department of Orthopedic Surgery, Zhongshan Hospital, Fudan University, Shanghai 200032, China; ^7^ Shanghai Engineering Research Center of Molecular Therapeutics and New Drug Development, College of Chemistry and Molecular Engineering, East China Normal University, Shanghai 200062, China

**Keywords:** GBM, glioma, proteomics analysis, gene expression analysis, Bioinformatics Analysis

## Abstract

Glioblastoma multiforme (GBM), the most aggressive and lethal primary brain tumor, is characterized by very low life expectancy. Understanding the genomic and proteogenomic characteristics of GBM is essential for devising better therapeutic approaches.Here, we performed proteomic profiling of 8 GBM and paired normal brain tissues. In parallel, comprehensive integrative genomic analysis of GBM was performed *in silico* using mRNA microarray and sequencing data. Two whole transcript expression profiling cohorts were used - a set of 3 normal brain tissues and 22 glioma tissue samples and a cohort of 5 normal brain tissues and 49 glioma tissue samples. A validation cohort included 529 GBM patients from The Cancer Genome Atlas datasets. We identified 36 molecules commonly changed at the level of the gene and protein, including up-regulated TGFBI and NES and down-regulated SNCA and HSPA12A. Single amino acid variant analysis identified 200 proteins with high mutation rates in GBM samples. We further identified 14 differentially expressed genes with high-level protein modification, among which NES and TNC showed differential expression at the protein level. Moreover, higher expression of NES and TNC mRNAs correlated with shorter overall survival, suggesting that these genes constitute potential biomarkers for GBM.

## INTRODUCTION

Glioma, which arises from glial cells, accounts for 80% of all malignant brain tumors [1]. The World Health Organization divides gliomas into four grades, of which glioblastoma multiforme (GBM) (grade IV) is the most aggressive and fatal. The median survival of GBM is approximately 15 months, even with standard-of-care treatment [2]. Therefore, it is vital to illuminate the molecular basis of GBM to develop effective therapies.

Recently, next-generation sequencing assays have provided platforms for identifying cancer susceptibility genes [3]. The cancer genome atlas (TCGA) has performed an integrative analysis of DNA copy number, gene expression and DNA methylation aberrations in GBMs [4]. However, the mRNA levels do not always correlate with the protein abundance, and the major proteome of GBM has not been fully elucidated [5, 6]. Thus, an integrated analysis of genomic and proteomic data may provide a more comprehensive understanding of the information flow that determines the GBM phenotype.

In this study, we performed label-free quantitative proteomics analysis for proteomic profiling and comprehensive integrative genomic analysis based on GEO and TCGA datasets of GBM to identify commonly changed molecular at the level of the gene and protein. Single amino acid variant analysis was also applied to identify proteins with high mutation rates in GBM samples. Our integrated analysis of the genomics and proteomics of GBM may provide a molecular basis and valuable insight for a new improved therapy of this disease.

## RESULTS

To systematically screen the mRNA levels of dysregulated genes in glioma development, we analyzed an Affymetrix U133 plus 2.0 microarray expression database containing 3 normal brain tissues and 22 glioma tissue samples, including 15 low grade glioma (grade I-II) and 7 GBM samples (GEO dataset: GSE45921). For low grade glioma compared to normal brain tissues, 492 genes displayed ≥ 2-fold difference at the *P* < 0.05 level and 45 genes displayed ≥ 5-fold difference at the *P* < 0.01 level (Supplementary Figure 1A, 1B) for GBM compared to normal brain tissues, 657 genes displayed ≥ 1.5-fold difference at the *P* < 0.05 level and 20 genes displayed ≥ 5-fold difference at the *P* < 0.01 level (Supplementary Figure 1C, 1D).

For verification, we also analyzed an BiostarH-140s × 32 microarray gene expression database with 3 normal brain tissues and 49 glioma tissue samples, including 22 low grade glioma and 27 GBM samples (GEO dataset: GSE51146). For low grade glioma compared to normal brain tissues, 399 genes displayed ≥ 2-fold difference at the *P* < 0.05 level and 21 genes displayed ≥ 5-fold difference in expression at the *P* < 0.01 level (Supplementary Figure 2A, 2B) for GBM compared to normal brain tissues, 609 genes displayed ≥ 1.5-fold difference in expression at the *P* < 0.05 level and 25 genes in 27 GBM tissue displayed ≥ 5-fold difference at the *P* < 0.01 level (Supplementary Figure 2C, 2D). Among the 609 genes in the GSE51146 dataset and the 657 genes in the GSE45921 dataset that displayed ≥ 1.5-fold difference in expression in GBM and normal brain tissue, there were 148 common differentially expressed genes.

**Figure 1:**
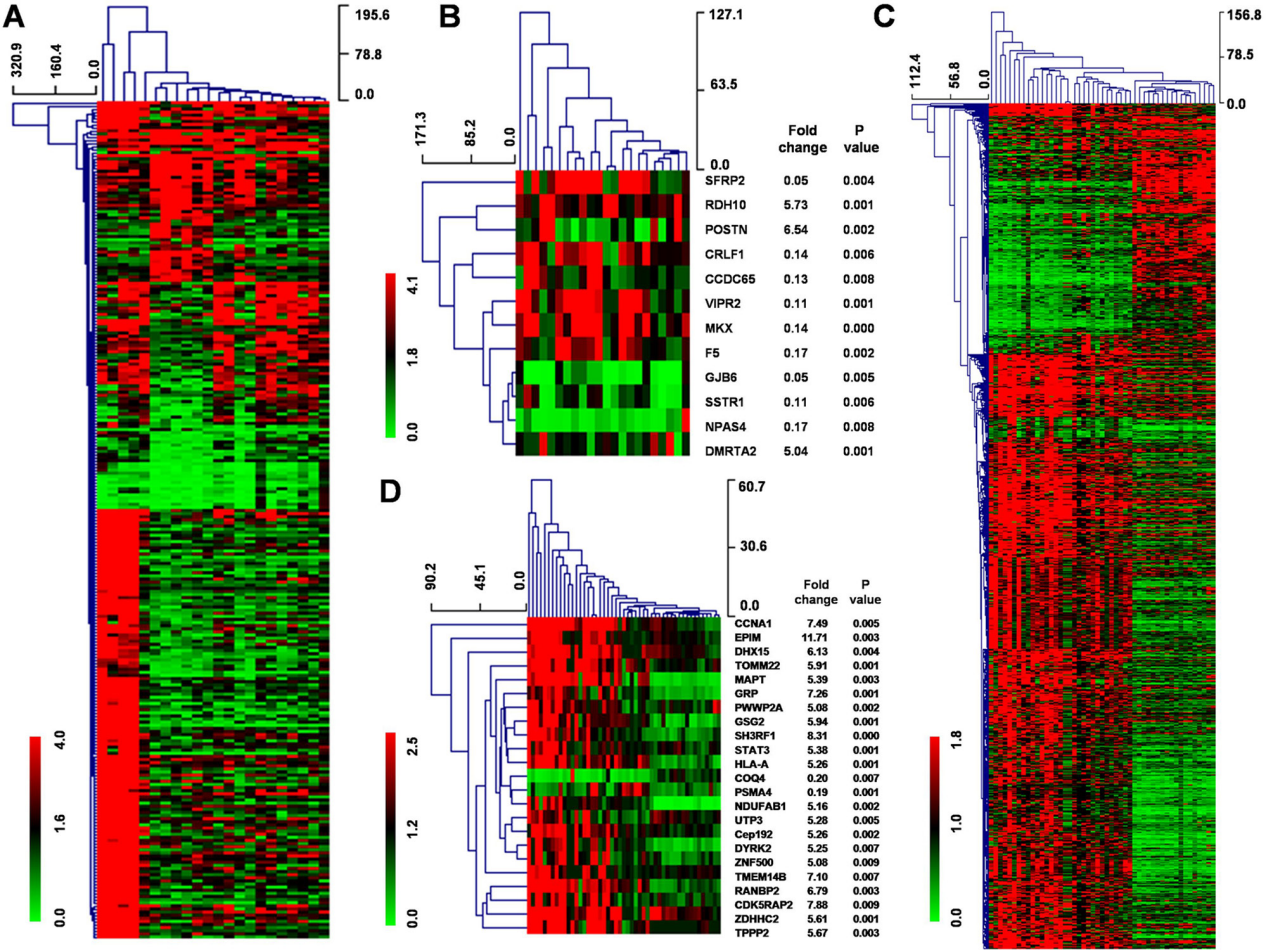
Hierarchal clustering of genes that are differentially expressed in low-grade glioma and GBM tissue **(A)** Hierarchical clustering for 269 differentially expressed genes (Fold change ≥ 2; *P* < 0.05) in 7 GBM tissues compared to 15 low-grade tissues using MEV4.7.1 software. **(B)** Hierarchical clustering for 12 differentially expressed genes (Fold change ≥ 5; *P* < 0.01) in 7 GBM tissues compared to 15 low-grade tissues using MEV4.7.1 software. **(C)** Hierarchical clustering for 136 differentially expressed genes (Fold change ≥ 2; *P* < 0.05) in 27 GBM tissues compared to 22 low-grade tissues using MEV4.7.1 software. **(D)** Hierarchical clustering for 23 differentially expressed genes (Fold change ≥ 5; *P* < 0.01) in 27 GBM tissues compared to 22 low-grade tissues using MEV4.7.1 software.

To examine stage-specific expression, we analyzed the differences between genes expressed in low-grade glioma and GBM tissues. For the GSE45921 database, 269 genes displayed ≥ 2-fold difference at the *P* < 0.05 level and 12 genes displayed ≥ 5-fold difference at the *P* < 0.01 level (Figure 1A, 1B). For the GSE51146 database, 136 genes in 27 GBM tissue displayed ≥ 2-fold difference at the *P* < 0.05 level and 23 genes displayed ≥ 5-fold difference at the *P* < 0.01 level (Figure 1C, 1D). Furthermore, there was little overlap in the genes that were expressed in low-grade glioma versus normal brain tissue and in GBM versus normal brain tissue in each of the data sets, suggesting that completely different biological processes mediate the development of low-grade glioma and GBM.

Next, we assessed Gene Ontology (GO) (Supplementary Figure 3A-3C), Kyoto Encyclopedia of Genes and Genomes (KEGG) pathways (Supplementary Figure 3D) and protein-protein interactions using STRING online analysis. Some functionally important proteins encoded by the 148 genes were identified, including polo like kinase 4 (PLK4), a tumor suppressor in GBM [7]; cyclin dependent kinase 6 (CDK6) [8]; PR/SET domain 10 (PRDM10) and myocyte enhancer factor 2C (MEF2C) (Supplementary Figure 3E).

Next, to further assess specific dysregulated protein in the occurrence and development of glioma, we performed LC-MS/MS-based whole proteomic profiling of 8 GBM and paired adjacent normal brain tissues in triplicate. In all, 693 proteins displayed ≥ 1.5-fold difference at the *P* < 0.05 level (Supplementary Figure 4A), and 15 significantly dysregulated proteins displayed ≥ 10-fold change at the *P* < 0.01 level (Supplementary Figure 4B). We performed GO (Supplementary Figure 4C-4E), KEGG pathway and protein-protein interaction analysis on the 693 differentially expressed proteins using STRING analysis. The top 5 KEGG pathways were oxidative phosphorylation, Huntington’s disease, Parkinson’s disease, Alzheimer’s disease, and metabolic pathways (Supplementary Figure 4F).

**Figure 2:**
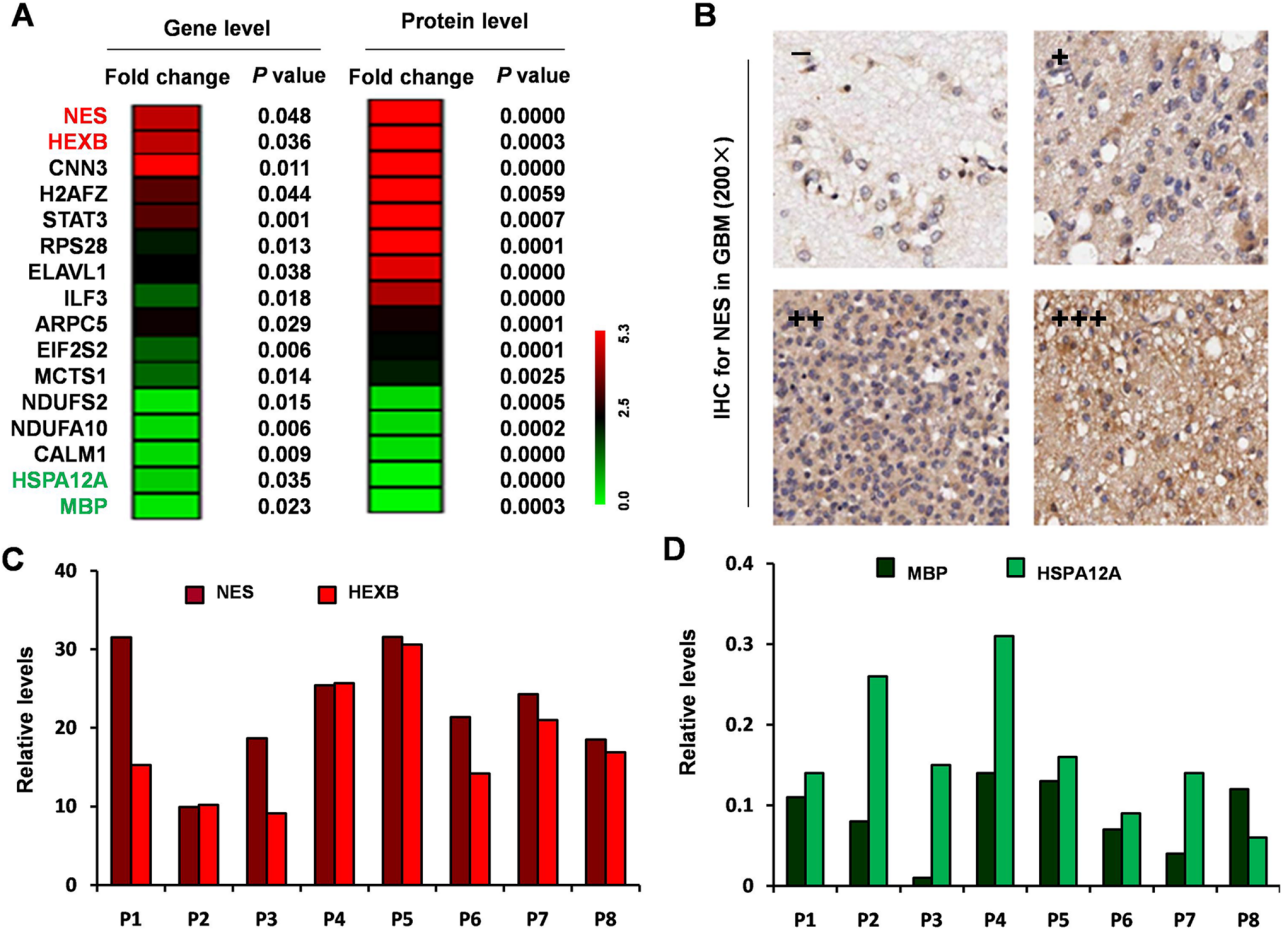
Integration of the differentially expressed proteins and genes from microarray **(A)** Heat maps for 16 common genes among the 148 differentially expressed genes that had similar expression patterns as corresponding proteins among the 693 differentially expressed proteins. **(B)** NES antibody staining of GBM tissues. **(C)** Relative expression level of NES and HEXB by qRT-PCR. **(D)** Relative expression level of HSPA12A and MBP by qRT-PCR.

To further evaluate whether the differential expression of the 693 proteins identified by LC-MS/MS proteins could be observed at the transcript level, we assessed overlap with mRNA expression. Sixteen of the 693 proteins were in common with the 148 GBM-specific genes identified by gene microarray analysis, (Figure 2A). We verified the consistent expression trend of the most significantly up-regulated (nestin [NES] and hexosaminidase subunit beta [HEXB]) and the most significantly down-regulated (heat shock protein family A member 12A [HSPA12A], myelin basic protein [MBP]) genes/proteins by IHC (Figure 2B) and qRT-PCR (Figure 2C, 2D).

**Figure 3:**
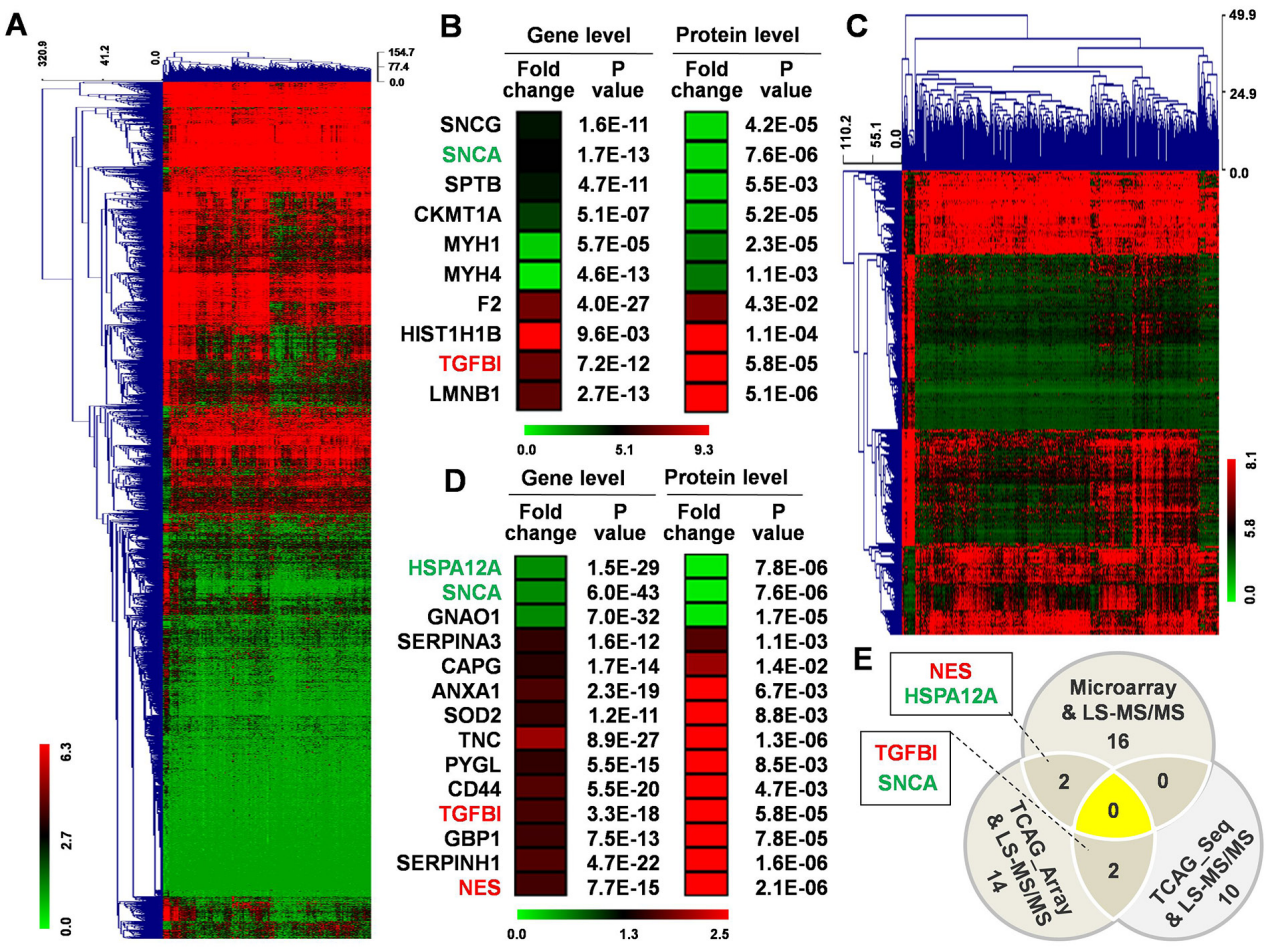
Analysis of TCGA datasets and comparison with protein expression data **(A)** Hierarchical clustering for 2881 differentially expressed genes (Fold change ≥ 1.5; *P* < 0.05) in 167 GBM tissue compared to normal brain tissue using MEV4.7.1 software. **(B)** 10 common genes contained among the 2881 differentially expressed genes and the 693 differentially expressed proteins. **(C)** Hierarchical clustering for 298 differentially expressed genes (Fold change ≥ 1.5; *P* < 0.05) in 529 GBM tissues compared to normal brain tissue using MEV4.7.1 software. **(D)** 14 common genes contained among the 298 differentially expressed genes and the 693 differentially expressed proteins. **(E)** 36 changed genes/proteins among three groups.

We also downloaded a GBM gene sequencing dataset (IlluminaHiSeq) from TCGA on the UCSC Genome Browser, which included 167 GBM and 5 normal brain tissue samples. There are 2881 genes differentially expressed in the GBM tissues (Fold change ≥ 1.5; *P* < 0.05) (Figure 3A), among which only 10 were in common with the 693 differentially expressed proteins identified in our study (Figure 3B).

In another GBM gene microarray dataset with 529 GBM and 10 normal brain tissue samples, 298 genes were differentially expressed in GBM tissues (Fold change ≥ 1.5; *P* < 0.05) (Figure 3C), with only 14 genes in common with our set of 693 differentially expressed proteins (Figure 3D).

Integrated analysis of the 693 GBM-specific proteins with the GBM-specific genes from 3 cohorts revealed a total of 36 differentially expressed genes/proteins (25 up-regulated and 11 down-regulated) (Figure 3E). Overlapping molecules in two groups included up-regulated transforming growth factor beta induced (TGFBI) [9] and Nestin (NES) [10]; and down-regulated synuclein alpha (SNCA) [11] and heat shock protein family A member 12A (HSPA12A), which have demonstrated roles in GBM and/or other cancers. These results suggest that the integrated analysis was effective in identifying potential molecular markers of GBM.

Furthermore, we performed GO (Supplementary Figure 5A-5C), KEGG pathway and protein-protein interaction analysis on the 36 cross-regulated genes/proteins. The top 5 KEGG pathways were Parkinson's disease, metabolic pathways, retrograde endocannabinoid signaling, oxidative phosphorylation, and neuroactive ligand-receptor interaction (Supplementary Figure 5D). Protein-protein interaction networks identified signal transducer and activator of transcription-3 (STAT3), ELAV like RNA binding protein 1 (ELAVL1), CD44 and calmodulin 1 (CALM1) (Supplementary Figure 5E).

**Figure 4:**
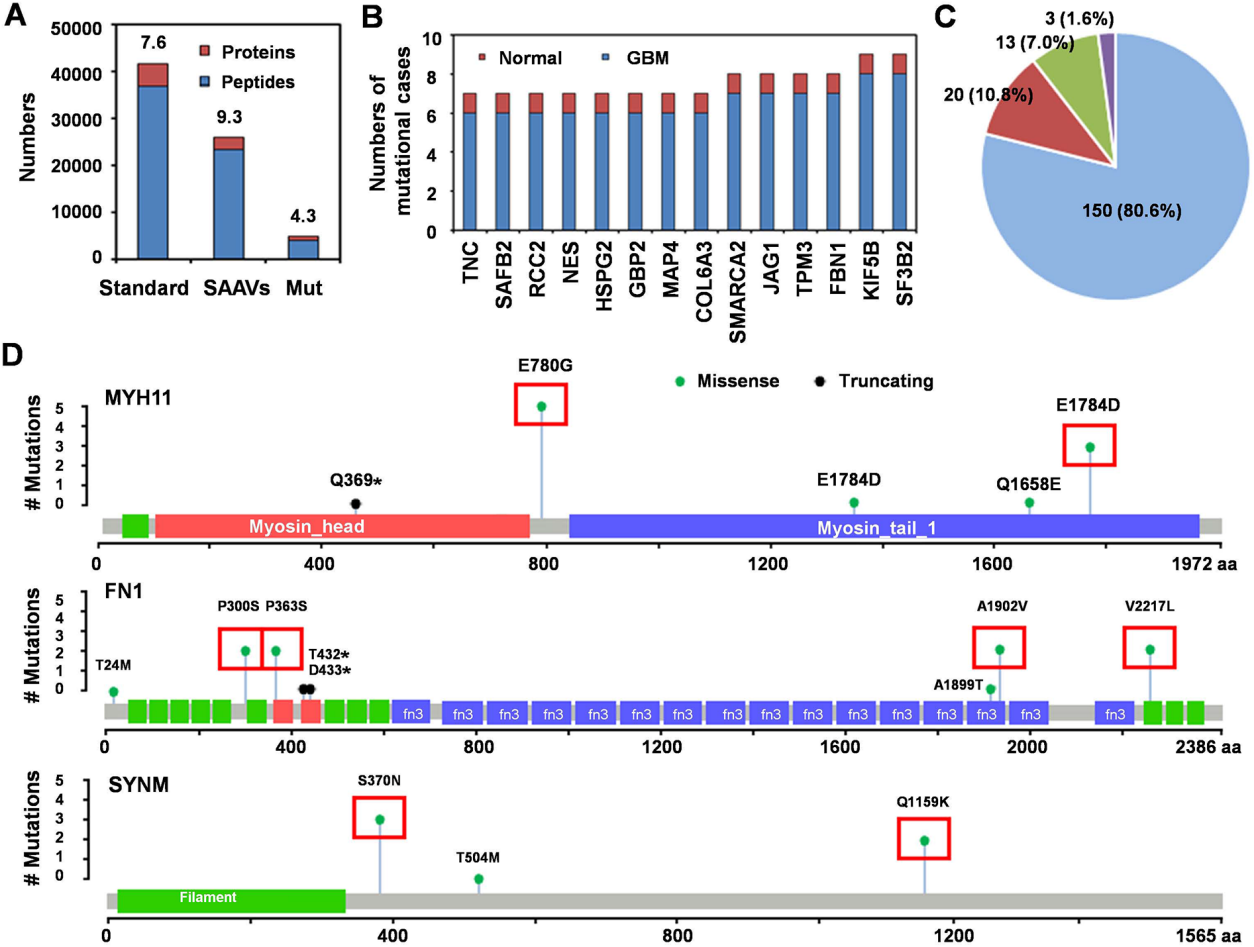
Single amino acid variants associated with GBM **(A)** the numbers of proteins and peptides in the standard protein library (Standard) and our customized SAAV database of GBM (SAAVs); and the numbers of mutated proteins/peptides uniquely identified in this study (Mut). Standard database included 36858 peptides in 4834 proteins; mean 7.6 peptides per protein and our customized mutation database included 23405 mutated peptides in 2515 proteins; mean 9.3 mutated peptides per protein. We identified 3884 peptide mutations in 897 proteins; mean 4.3 mutated peptides per protein. **(B)** the number of samples with mutations in 14 peptides of 14 proteins in GBM and normal brain samples with 75-99% mutation rate. **(C)** the percent of proteins with different mutation numbers among those with SAAVs that were exclusively observed in GBM (100% mutation rate). Blue, 1 SAAV; red, 2 SAAVs; green, 2-4 SAAVs; and purple, more than 4 SAAVs. **(D)** the number of mutation sites and cases of GBM for the 3 most frequently mutated proteins (MYH11, FN1 and SYNM). Red boxes indicate the mutation site and mutation cases in 8 GBM patients.

As variant peptides are not included in the standard UniProt Human database (36858 peptides in 4834 proteins), we created a customized mutation database (23405 mutated peptides in 2515 proteins; mean 9.3 mutated peptides per protein) to identify single amino acid variants (SAAVs). Our results revealed 3884 peptide mutation in 897 proteins (mean 4.3 mutated peptides per protein) (Figure 4A). Fourteen peptides of 14 proteins were mutated only in 1 of 8 normal brain tissues (mutation rate 12.5%) but in ≥ 6 of 8 GBM tissues (mutation rate 75%) (Figure 4B). Additionally, 335 peptides of 186 proteins were mutated exclusively in GBM tissue (mutation rate 100%), of which, 150 (80.6%) had only a single SAAV, 20 (10.8%) had 2 SAAVs, 13 (7.0%) had 2-10 SAAVs and 3 (1.6%) had more than 10 SAAVs (MYH11, 48 SAAVs; FN1, 16 SAAVs; SYNM, 14 SAAVs) (Figure 4C-4D).

**Figure 5:**
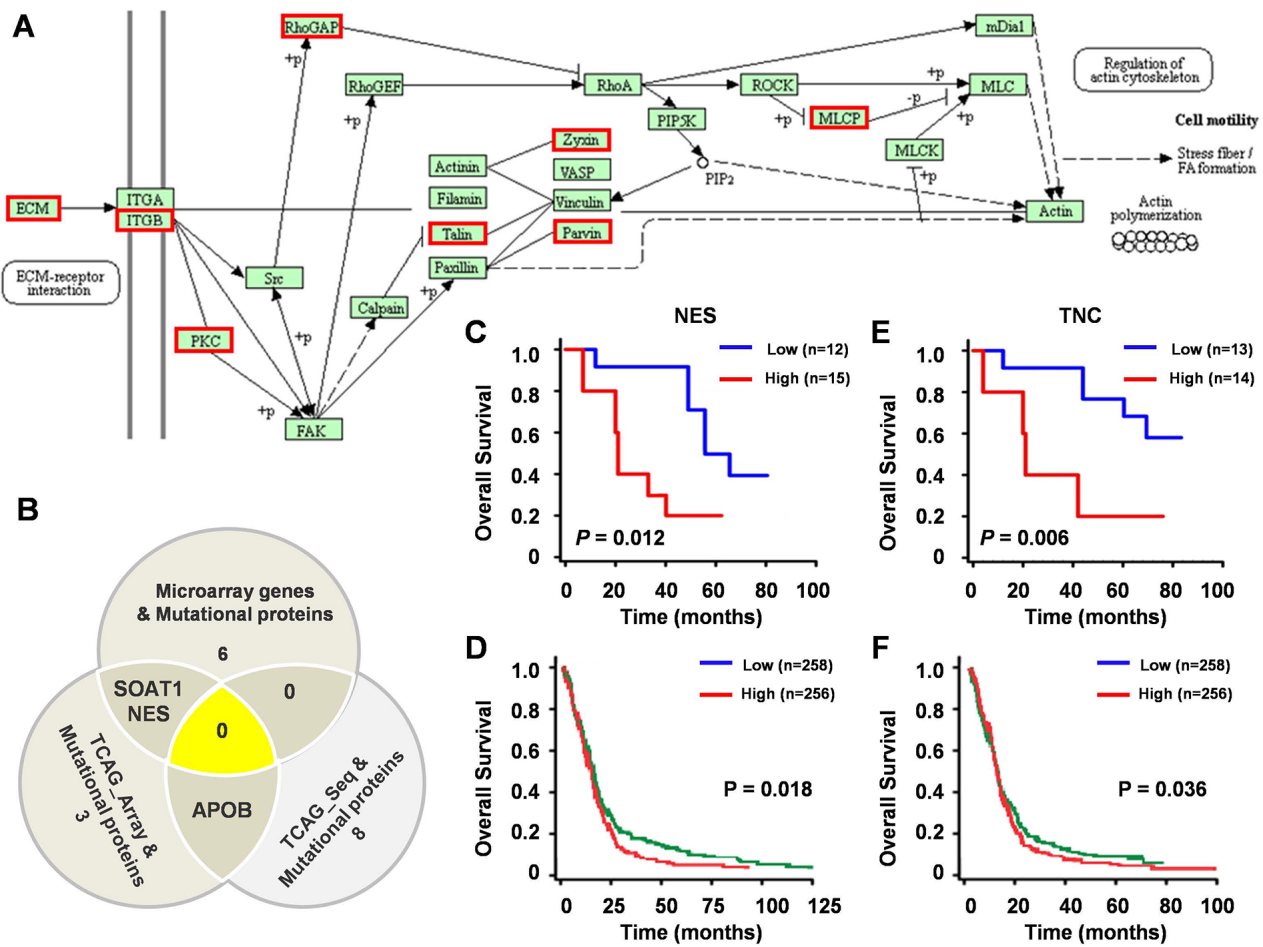
Clinical significant of mutated proteins **(A)** 19 proteins (9.5%) enriched in the Focal adhesion pathway by KEGG pathways classification enrichment analysis. Red boxes indicate the proteins in the Focal adhesion pathway that were mutated in GBM. **(B)** 14 commonly identified genes among three groups. SOAT1, NES and APOB were identified in more than one group. Univariate analysis of OS and expression of NES mRNA in 27 GBM patients **(C)** and 514 GBM patients **(D)** Univariate analysis of OS and expression of TNC mRNA in 27 GBM patients **(E)** and 514 GBM patients **(F)**.

KEGG pathway enrichment analysis for the 200 proteins with ≥ 75% mutation rate indicated that 19 proteins (9.5%) were enriched in the “Focal adhesion pathway” (Figure 5A). Additionally, 6 variant proteins were represented within the 148 differentially expressed genes from microarray, 3 were represented within the 298 differentially expressed genes from TCGA array, and 8 were represented within the 2881 differentially expressed genes from TCGA sequencing (Figure 5B). Two of the 14 molecules (NES and TNC) also showed differential protein expression in the LS-MS/MS assay.

We analyzed the clinical significance of NES and TNC using data of 529 GBM patients from TCGA datasets and 27 GBM patients from our gene expression array. High expression of NES (Figure 5C, 5D) and TNC (Figure 5E, 5F) correlated with shorter overall survival, suggesting that these two molecules are potential biomarkers for GBM, suggesting that these two molecules constitute potential biomarkers for GBM.

## DISCUSSION

GBM is the most aggressive and lethal primary brain tumors, characterized by very low life expectancy. In this study, we present an integrated study of genomic and proteomics in GBM and paired normal brain tissues, which provides a number of insights into the biology of GBM and identifies potential therapeutic targets.

Through gene expression analysis based on GEO datasets and TCGA datasets and proteomic analysis, we identified 36 molecules commonly changed at the level of the gene and protein. Our results revealed that mRNA transcript abundance could relatively reliably predict protein abundance differences. Interestingly, we found that many transcripts are expressed differentially between the low-grade glioma and GBM tissue samples. Our results also suggest that distinct biological process may characterize different stages of glioma.

In this study, several of the differentially expressed molecules identified, including TGFBI, NES, SNCA, and HSPA12A, have demonstrated roles in GBM and other cancers. TGFBI encodes an RGD-containing protein that binds type I, II and IV collagens and is functionally up-regulated in GBM upon TGF-beta signaling [9]. NES, which encodes a member of the intermediate filament protein family, is expressed primarily in nerve cells and is a prognostic marker in GBM [10]. Alpha-synuclein is a member of the synuclein family, which is abundantly expressed in the brain; its overexpression increased the vulnerability of U373 cells to apoptosis through TNF-alpha-mediated pathways [11]. Furthermore, our result of proteins and pathways identified by GO and KEGG, including STAT3and CD44, were in agreement with that of previous reports, which verified their function in GBM. STAT3 has been clearly implicated in the development, progression, and aggressiveness of GBM [12]. CD44, a transmembrane molecule with several isoforms, is overexpressed in many tumors, including GBM and has been implicated in malignant processes including cell motility, tumor growth, and angiogenesis [13]. Until now, clinical trials investigating CD44 targeting in CD44-positive solid tumors are underway, and the evidence presented in the previous report suggests that CD44 inhibition in GBM may be a promising therapy.

A fundamental question in proteogenomics is to identify expressed protein coding alterations at the protein level. However, standard database search approaches cannot identify variant peptides from MS/MS data. We created a customized mutation database of GBM to performed database searches for SAAVs from the COSMIC database and performed database searches with customized sequence databases to identify SAAVs. Our SAAVs analysis identified 200 proteins with high mutation rates in GBM samples. To better understand the biology underlying the proteomic variants, we further identified 14 differentially expressed genes with high-level protein modification, among which NES and TNC showed differential expression at the protein level. Moreover, higher expression of NES and TNC mRNAs correlated with shorter overall survival, which suggesting that cancer-related variant proteins may serve as candidate protein biomarkers or therapeutic targets.

Taken together, our proteomic characterization of the genomically annotated GBM clarifies the power of integrated proteomics and genomics analysis and provides a molecular basis and valuable insight for new advances in GBM therapy. Moreover, our protein coding alteration approach provided new insights into their roles in GBM and may be broadly extended to understand roles of protein mutation in other cancers.

## MATERIALS AND METHODS

### Tissue samples and ethics statement

The study protocol and acquisition of tissue specimens were approved by the Ethical Committee of Shanghai Tenth People’s Hospital, Tongji University School of Medicine, and Ganzhou City People’s Hospital (2015-Res-10). This study was registered with ClinicalTrials.gov, number NCT01454102. Each participant provided written informed consent before participating in this study. We collected 8 GBM samples and paired normal brain tissue from patients undergoing surgical resection and classified according to the last WHO classification of central nervous tumors, which was confirmed by two experienced pathologists independently.

### RNA extraction

According to the manufacturer’s guideline, total RNA was isolated using Trizol regent (Invitrogen, Carlsbad, CA, USA). RNA quantity was determined using NanoDrop ND-1000 spectrophotometer and the integrity of RNA was measured by gel electrophoresis.

### Real-time quantitative reverse transcription PCR

cDNA was synthesized from total RNA (10 ng), and quantitative PCR reactions were performed with the Taqman™ Universal PCR Kit (Life Technologies). GAPDH was used as the internal control, and the 2^-ΔΔCT^ method was used to analyze the expression levels of genes.

### Immunocytochemistry

GBM paraffin sections were cut into 4-μm thick sections, then added onto poly-lysine coated slides and incubated at 65°C overnight. The incubated slides were then deparaffinized in xylene and rehydrated with graded alcohol. Next, retrieve the antigen using citrate buffer (pH 6.0) and store the slides in Tris buffered saline. In order to block endogenous peroxidase activity, 3% hydrogen peroxide was added to the slides. They were then incubated overnight at 4°C in monoclonal antibody (Novus, Littleton, CO, USA) solution at 1:200 dilution. Finally, the slides were incubated with horseradish peroxidase-conjugated goat anti-rabbit immunoglobulin, and color was developed using the DAB Horseradish Pe-roxidase Color Development Kit (Maixin Co., Fuzhou, China).

### Protein extraction and analysis by nano-LC-MS/MS

Paired 8 GBM and normal brain tissues were cut into small pieces (about 1 mm^3^) and rinsed in PBS to remove blood. Then tissue were homogenized in 4% SDS and 0.1 M DTT in 0.1M Tris-HCl, pH7.6 solution on ice, sonicated 10 times (80 w, working 10 s, suspending 15 s), incubated for 5 min at 95 °C. The protein concentrations of clarified lysates were determined using fluorescence assay. A 200 μg of clarified lysates were proteolysedon 10 kDa Filter (PALL Life Sciences, USA) using a Filter Aided Sample Preparation (FASP) method. The peptide samples were then desalted onto a solid-phase extraction cartridge (Empore 7 mm/3 ml). The lyophilized peptide mixture was re-suspended in water with 0.1% formic acid (v/v) and its content was estimated by UV light spectral density at 280 nm, then 3 μg of the digest peptides were analyzed by nano-liquid chromatography-tandem mass spectrometry (LC-MS/MS) on LTQ Orbitrap Velos Pro mass spectrometer [14].

Raw data were processed by by Maxquant software (1.3) and then used for database and spectral library searching using Andromeda peptide search engines. The Maxquant peptide and protein quantification results files were imported into Perseus software (version 1.5.1.6) for further analysis. All of the MS proteomics data have been deposited to iProX (http://www.iprox.org/index) and can be accessed with the accession IPX00084901.

### Bioinformatic analysis

The expression levels of genes were investigated in paired GBM tissue samples based on GEO datasets (GSE45921 and GSE51146) using the NCBI Platform (http://www.ncbi.nlm.nih.gov/) and TCGA datasets (TCGA-IlluminaHiSeq and TCGA_GBM_Exp_U133a) from UCSC Genome Browser (https://genome-cancer.ucsc.edu/). Hierarchical clustering was performed using the multiple experiment viewer (MeV) 4.7.1 software (http://www.tm4.org/mev/).

We used GO classifications (http://www.geneontology.org/) to evaluate the biological function of the changed genes in GBM through three aspects including biological process, molecular function and cellular components. Subsequently, dysregulated genes were subjected to KEGG pathway analysis. Protein-protein interaction networks analysis was performed by STRING (http://string-db.org/).

### Statistical analysis

The results were expressed as mean ± S.D. (standard deviation). Statistical significance between the groups was assessed by using one-way analysis of variance (ANOVA). Univariate survival analysis and multivariate analyses were carried out using the Kaplan–Meier method. All calculations were performed with the SPSS 20.0 software program (SPSS Inc, Chicago, IL, USA). The level of significance was chosen as *P* < 0.05.

## SUPPLEMENTARY MATERIALS FIGURES



## Abbreviations

GBM: Glioblastoma multiforme
GO: Gene Ontology
KEGG: Kyoto Encyclopedia of Genes and Genomes
LC-MS/MS: liquid chromatography-tandem mass spectrometry
SAAVs: single amino acid variants
TCGA: The cancer genome atlas
