# Supplementary material for: Proteogenomic characterization and integrative analysis of glioblastoma multiforme
Source: Oncotarget. 2017 Oct 19;8(57):97304–12. doi: 10.18632/oncotarget.21937 (PMC5722563; doi:10.18632/oncotarget.21937)
Supplement: Supplementary file 1 [file oncotarget-08-97304-s001.pdf]

Proteogenomic characterization and integrative analysis of glioblastoma multiforme

SUPPLEMENTARY MATERIALS

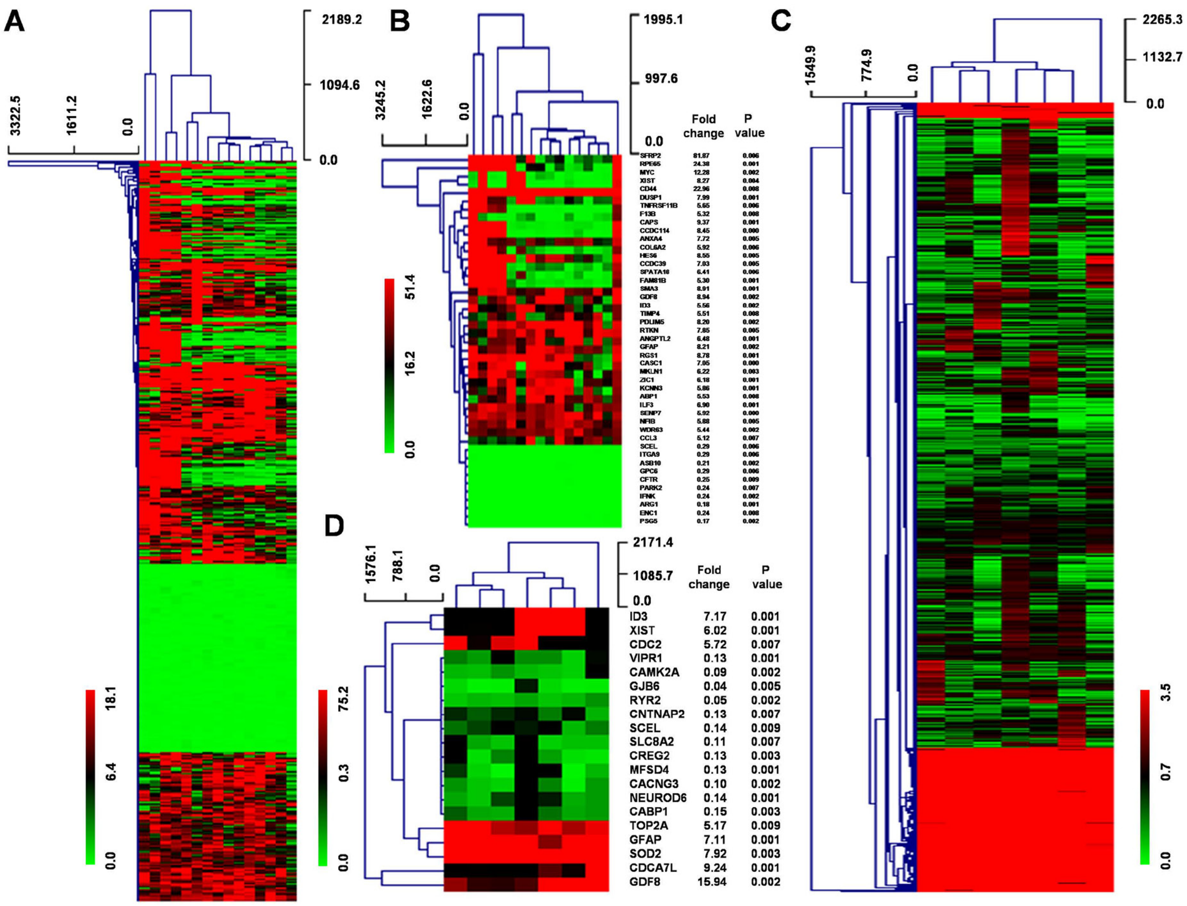

Supplementary Figure 1: Hierarchal clustering of gene expression data based on Affymetrix U133 plus 2. 0 microarrays. **(A)** Hierarchical clustering for 492 differentially expressed genes (Fold change ≥ 2; $P < 0.05$) in 15 low grade glioma tissues using MEV4.7.1 software. **(B)** Hierarchical clustering for 45 differentially expressed genes (Fold change ≥ 5; $P < 0.01$) in 15 low grade glioma tissues using MEV4.7.1 software. **(C)** Hierarchical clustering for 657 differentially expressed genes (Fold change ≥ 1.5; $P < 0.05$) in 7 GBM tissues using MEV4.7.1 software. **(D)** Hierarchical clustering for 20 differentially expressed genes (Fold change ≥ 5; $P < 0.01$) in 7 GBM tissues using MEV4.7.1 software.

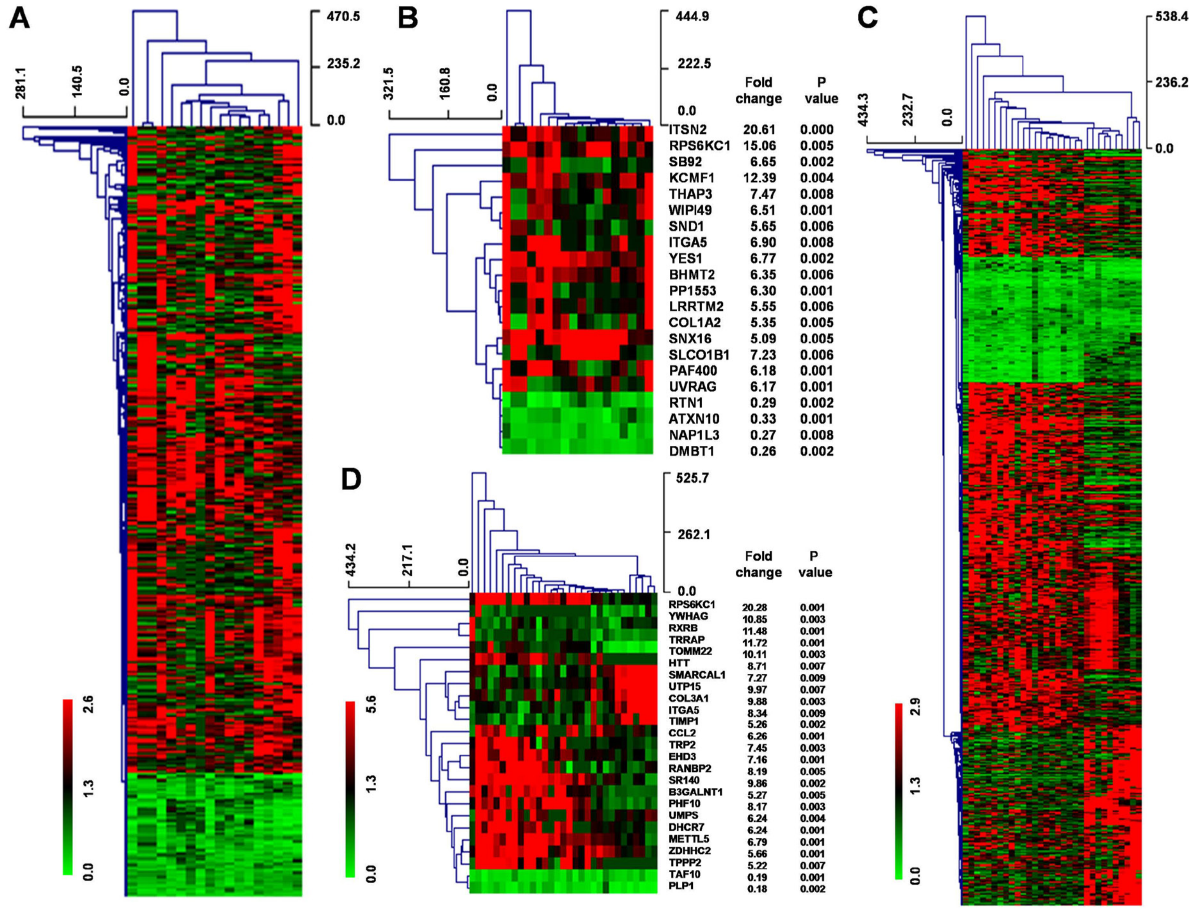

Supplementary Figure 2: Hierarchal clustering of gene expression data based on BiostarH-140s × 32 microarrays. (A) Hierarchical clustering for 399 differentially expressed genes (Fold change ≥ 2; $P < 0.05$) in 22 low grade glioma tissues using MEV4.7.1 software. **(B)** Hierarchical clustering for 21 differentially expressed genes (Fold change ≥ 5; $P < 0.01$) in 22 low grade glioma tissues using MEV4.7.1 software. **(C)** Hierarchical clustering for 609 differentially expressed genes (Fold change ≥ 1.5; $P < 0.05$) in 27 GBM tissues using MEV4.7.1 software. **(D)** Hierarchical clustering for 25 differentially expressed genes (Fold change ≥ 5; $P < 0.01$) in 27 GBM tissues using MEV4.7.1 software.

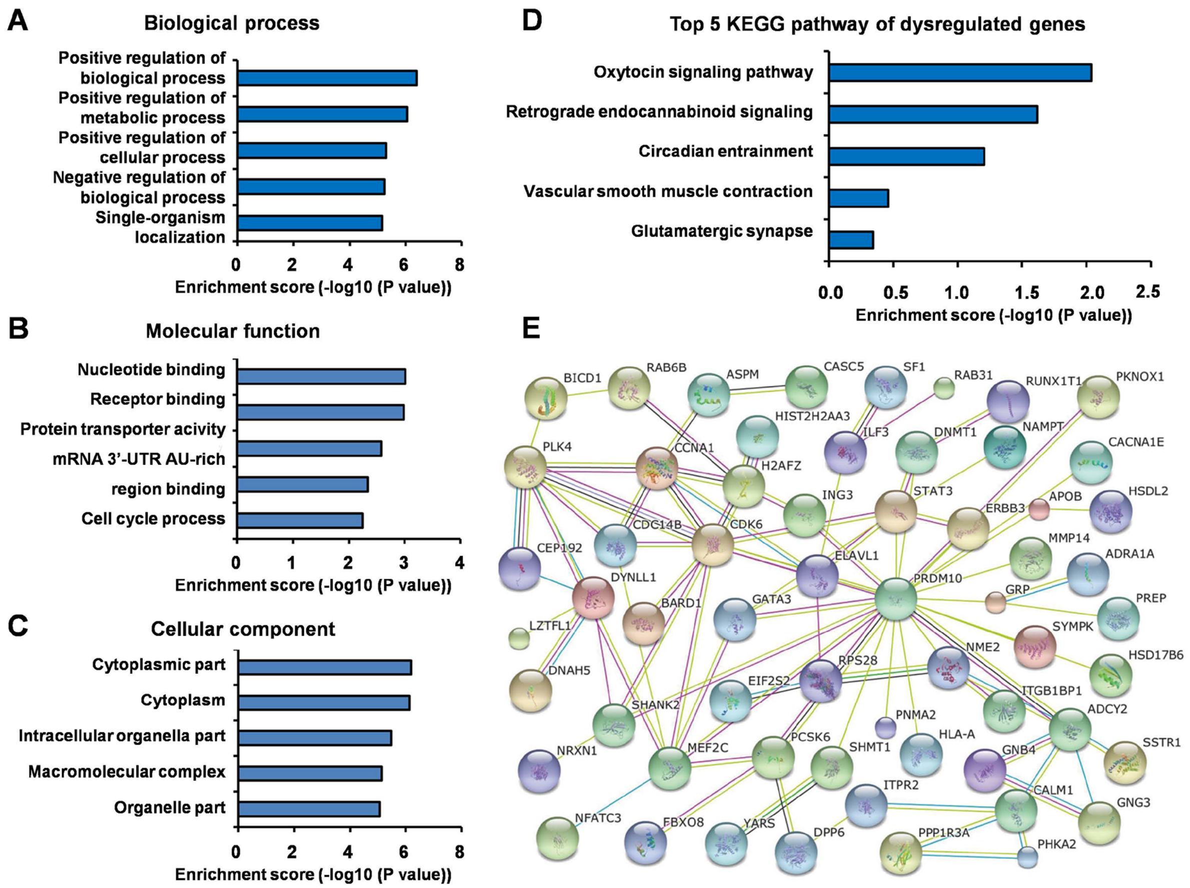

Supplementary Figure 3: GO, KEGG pathway and Protein-Protein Interaction analysis of 148 genes. (A-C) The top 5 GO terms under “Biological process”. (A), “Molecular function” (B) and “Cellular component” (C) for the 148 differentially expressed genes that were identified in 3 different data sets. **(D)** The top 5 KEGG pathways for the 148 differentially expressed genes. **(E)** Protein-protein interaction networks analysis for the 148 differentially expressed genes.

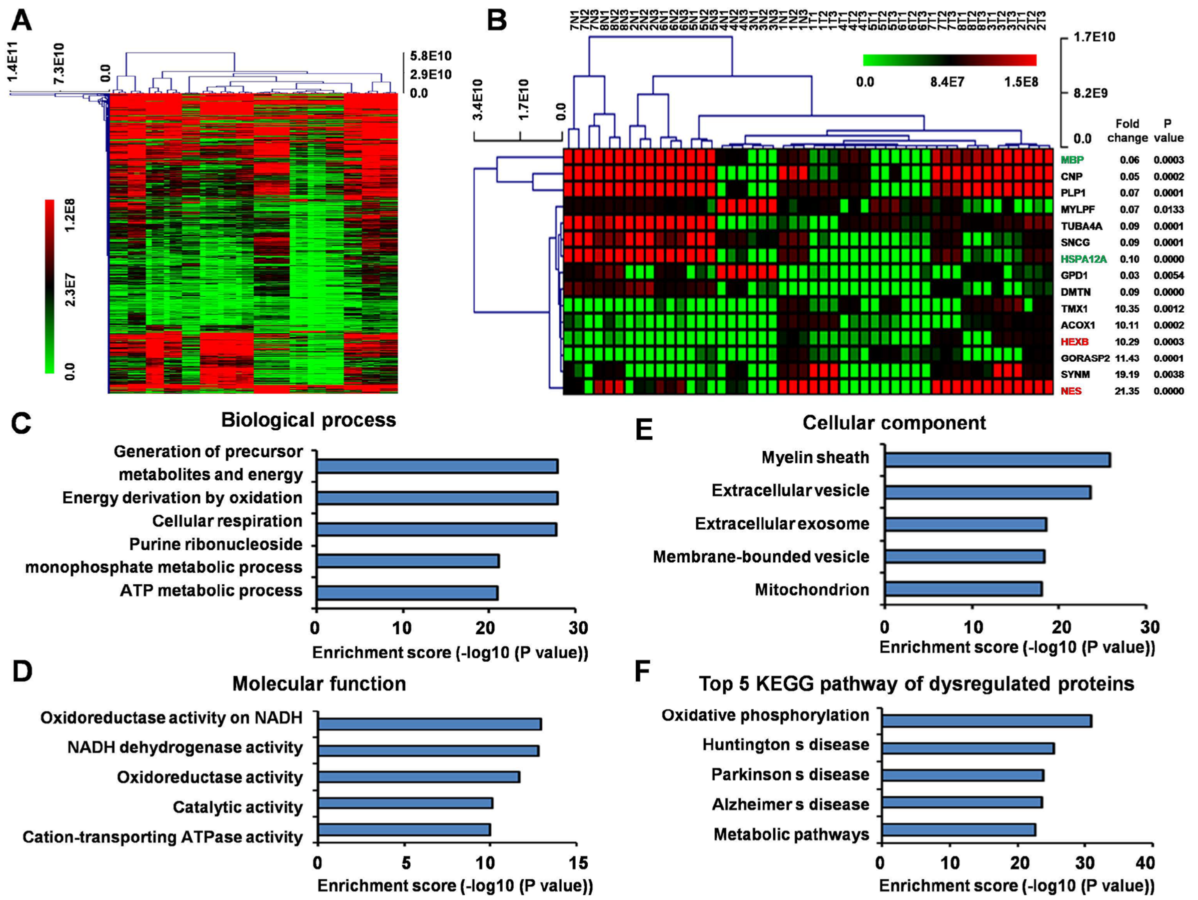

Supplementary Figure 4: Proteomic analysis of 8 paired GBM tissue. **(A)** Hierarchical clustering for 693 differentially expressed proteins (Fold change ≥ 1.5; $P < 0.05$) in 8 GBM tissues. **(B)** Hierarchical clustering for 15 significant differentially expressed proteins (Fold change ≥ 10; $P < 0.01$) in the 8 GBM tissues. **(C-E)** The top 5 GO terms under "Biological process" (C) "Molecular function" (D) and "Cellular component" (E) for the 693 differentially expressed proteins. **(F)** The top 5 KEGG pathways of the 693 differentially expressed proteins.

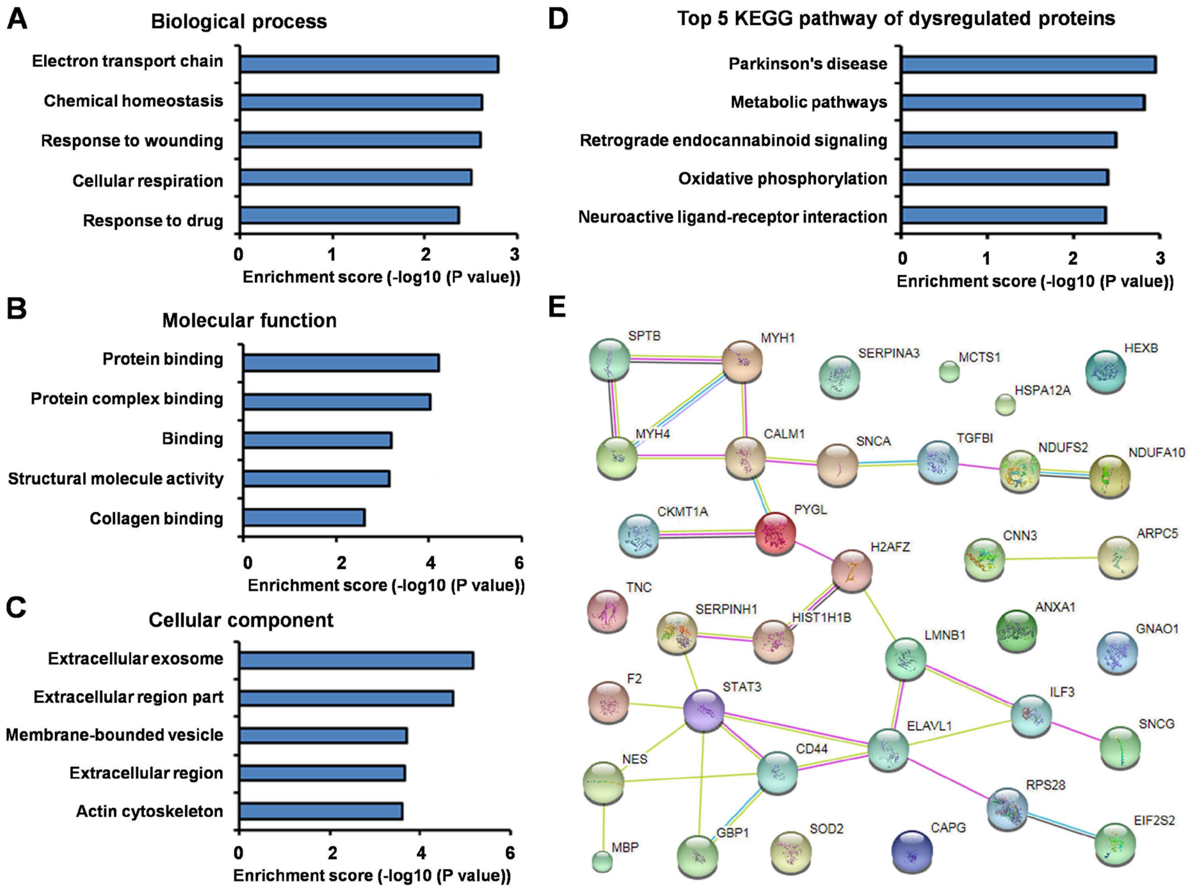

Supplementary Figure 5: GO, KEGG pathway and Protein-Protein Interaction analysis of 36 genes. (A-C) The top 5 GO terms under "Biological process" (A), "Molecular function" (B), and "Cellular component" (C) for the 36 differentially expressed genes. **(D)** The top 5 KEGG pathway for the 36 differentia lly expressed genes. **(E)** Protein-protein interaction networks analysis for the 36 differentially expressed genes.
